# A Review of Sensorineural Hearing Loss in Congenital Cytomegalovirus Infection

**DOI:** 10.7759/cureus.30703

**Published:** 2022-10-26

**Authors:** Garima Singh, Abhay Gaidhane

**Affiliations:** 1 Community Medicine, Jawaharlal Nehru Medical College, Datta Meghe Institute of Medical Sciences, Wardha, IND

**Keywords:** vertical transmission, sensorineural hearing loss, hearing impairment, congenital infection, cytomegalovirus

## Abstract

The intrauterine transmission of the TORCH group (toxoplasmosis, rubella cytomegalovirus (CMV), herpes simplex, and HIV) produces severe complications in the fetus, leading to major life challenges for the newborns. CMV is considered one of the leading causes of congenital hearing loss in babies born to infected mothers. The majority of the cases are asymptomatic but a certain proportion show symptoms, which may not be present until later. The human cytomegalovirus (HCMV) infection can also be transferred to the baby postnatally through breastfeeding. Mothers facing primary infection from the CMV have a greater tendency to transmit the infection to the fetus, whereas secondary infection due to reactivation of the virus in females who were affected before pregnancy, delivers asymptomatic babies in most cases. Vertical transmission of HCMV, that is via the placenta from infected mother to fetus, is one of the leading causes of congenital sensorineural hearing loss (SNHL) and neurodevelopmental complications in newborns. It accounts for up to 10% of SNHL cases in newborns, of both unilateral and bilateral types. Antiviral therapy is helpful in such cases if administered within the first month of life, and for hearing impairment, cochlear implants have been used to treat children who develop profound hearing loss. The child can present with hearing loss at birth or it may be late in onset and of progressive type. Suspected children should be evaluated regularly for the early detection of hearing loss and to provide the appropriate treatment.

## Introduction and background

Cytomegalovirus (CMV) infection is one of the most common intrauterine infections. CMV can be found everywhere due to ineffective strategies to prevent its infection. People affected may or may not show symptoms. It can be transmitted through body fluids. Humans are the primary reservoirs. The virus is shed in the body fluids such as saliva, urine, blood, semen, etc., of the infected individual. About 0.6%-6.1% of babies are born with congenital CMV infection each year [1]. People who are in regular contact with children (i.e. daycare workers, teachers, and play school staff) are at a higher risk of acquiring CMV by coming in contact with body fluids of the baby like saliva, urine, or coming in contact with baby toys, diapers, etc. CMV-infected children under two years of age secrete viruses in their urine and saliva for about 24 months [2]. Once the virus enters the body, it remains there for life. There is no known cure for CMV infection. The virus may be present in an inactive form but might also get reactivated later in life. The majority of the people in the world are infected with CMV, but only the ones with weak immune responses show up with symptoms. The other asymptomatic cases go unnoticed.

Approximately 1-4 out of every 100 females are affected by CMV infection for the first time during pregnancy [3]. Conditions like immune compromise and organ or bone marrow transplant or continuous immunosuppressant medications increase the risk of symptoms and complications from CMV infection [4]. The hormonal changes happening during pregnancy can increase the risk that a pregnant woman is affected by CMV. If a female has been infected with CMV before pregnancy, it may reactivate, resulting in vertical transmission to the fetus. There is also a risk that someone who has previously been infected with the CMV may become infected with a different strain. Pregnant females with primary CMV infections have a 30%-40% chance of transmitting the infection to the baby; if the female gets infected in the third trimester of her pregnancy, then there are 40%-70% chance of the baby getting infected [5]. The baby can also be infected via breast milk if the mother is infected after delivery.

Universal testing of CMV during pregnancy is not recommended currently but is advised to be done in some cases, which include illness presenting like mononucleosis if the pregnant woman comes in contact with someone already having CMV infection, occupational exposures in case of healthcare or childcare worker, or if fetal sonography is suggestive of congenital CMV. An infected person will present with complaints like fever, exhaustion, swollen glands, loss of appetite, muscle loss, stiff joints, and weakness. Clinical manifestations of the primary CMV infection are similar to the infection caused due to the Epstein-Barr virus and present with fever, malaise, pharyngitis, headache, hepatosplenomegaly, arthralgia, and rash. Blood tests can be performed for CMV antibodies to diagnose the infection in previously affected mothers; the blood sample will show antibodies against the CMV. Amniocentesis can be done to check for infection in the fetal blood sample, but amniocentesis can still be risky as this might transmit the infection if it wasn't transmitted to the fetus yet. Once the infection has been transmitted to the fetus, there are several manifestations of the infection. Even though about 10%-15% of the asymptomatic infants experience some or the other type of permanent sequelae, the symptomatic group of babies will present with manifestations like rashes, yellowing of skin or sclera of the eyes, microcephaly, low birth weight, retinitis, hepatosplenomegaly and seizures at the time of birth. Fetal ultrasound showing intracranial calcification, hydrops, hyperechogenic bowel [6], presence of ventriculomegaly, occipital horn abnormalities, pericardial effusion, ascites, intrauterine growth restriction, etc. is suggestive of infection with congenital CMV infection. Placental inflammation and fetal death are also seen [7-8]. Postnatally the tests that can determine whether the baby is infected with CMV are urine or saliva polymerase chain reaction (PCR) tests as there is shedding of the virus in the body fluids of the baby.

## Review

Sensorineural hearing loss in congenital CMV infection

Hearing loss is the hallmark of congenital CMV infection. CMV is also the most common non-genetic cause of sensorineural hearing loss in newborns [9]. Even if the mothers are seroimmune, babies may still develop hearing loss. The hearing impairment may or may not be present at the time of birth and may appear later in life. But it is the most common manifestation of CMV infection in newborns. This type of hearing loss is late in onset and progressive in nature, and the hearing loss progresses through adolescence. Infants born to seroimmune mothers experience a milder form of hearing loss. The infants showing symptoms of the CMV infection have a greater degree of severity of hearing loss in comparison to the asymptomatic ones. Another thing to be noted in case of hearing loss associated with CMV is fluctuating hearing which is unexplained by concurrent middle ear infections. By this, we mean that it may seem to occur in one ear at only a few or specific frequencies or in both ears in case of bilateral hearing loss. The hearing loss due to CMV can be either unilateral or bilateral types.

Signs and symptoms

The signs and symptoms of hearing loss in babies can be seen if the baby does not startle even in loud noises and does not respond or turn to the source from where the sound is coming. Even after six months of age, if the child is unable to speak single words such as mama or dada, till he/she reaches one year of age. Or the baby hears some sounds and does not respond to them, he/she turns around only if they see you and not when you call them or react to noises or voices. Signs of hearing loss include delayed speech: the speech isn't clear, isn't able to follow directions which can be a sign of partial hearing loss, often questions back by saying 'huh?', watches television at a very high volume, etc. [10].

The pathological findings showed inclusion bodies typical of CMV, i.e., multiple vacuoles including clusters of the viral particles in the nucleus of the affected cells of the endolymphatic sac epithelium, the semicircular canals, and the utricle. Loss of the inner and outer hair cells, as well as cochlear ganglion cells, was also seen [11]. Numerous ear conditions, including otosclerosis [12], Meniere's disease [13], and other types of sensorineural hearing loss, have been theorised to have a viral genesis. Immune-mediated inner ear illness may be caused by CMV damage, which may result in endolymphatic hydrops [14]. It is plausible that CMV infection causes tinnitus and a pro-inflammatory state called sensorineural hearing loss [15]. The onset of deafness in children with congenital CMV infection can happen as early as 6 to 8 years of age [14].

Mechanism

Even though the exact mechanism is unknown, two potential pathways could explain the clinical history of the progressive type of hearing loss caused by infection due to the CMV, the direct harm caused to the spiral ganglion neurons or the hair cells by an ongoing CMV infection and CMV damage caused to the infected cells by host immune system [16]. The discovery of DNA of the CMV in children's inner ear secretions led researchers to hypothesise that the CMV not only damages the inner ear when it is contracted during pregnancy and the perinatal period but also later in life. The disruption of potassium homeostasis caused by injury to the Reissner's membrane or stria vascularis with subsequent damage to the organ of Corti may be the primary mechanism of sensorineural hearing loss in CMV congenital infection. As the virus favours the undifferentiated cells, this mechanism can also be proposed for non-congenital infection (such as stem cells) [17-18]. The inner ear's injury mechanism can be affected by transient changes in cell-mediated immunity due to concomitant viral infections and the pathogenicity of CMV. For the virus to be defined as the causative agent of hearing loss, these three criteria must be met: (1) if the virus can be isolated from the external or internal ear successfully, (2) morphological changes in the cochlear cells due to the viral infection, and (3) viral antigens detected in the cells of the cochlea by the immunohistochemical analysis [19]. It is difficult to find out whether these three criteria are fulfilled or not because of the dense lining of the temporal bone in the inner ear.

Treatment

Antiviral therapy for CMV infection has been useful in preventing the virus's major effects. Many newborns who receive treatment with valganciclovir or ganciclovir may see improvement in their mild or moderate hearing loss [20-25]. Symptomatic newborns affected with the CMV should be administered oral valganciclovir or ganciclovir for six months. These drugs inhibit the replication of the virus by disrupting their mechanism of viral DNA synthesis [26]. Ganciclovir was the first ever drug to be used for the treatment of human cytomegalovirus (HCMV) infections. It has been found to be safe, effective, and tolerable for severe organ diseases. Valganciclovir being the prodrug of ganciclovir, is effective enough just like intravenous ganciclovir with lesser short-term adverse effects [27]. This method of treatment has resulted in the prevention of the progression of hearing loss or reduced its effect to a great extent. It also improves other neurological and developmental conditions affected because of the infection [28]. Ganciclovir might lead to neutropenia or some other toxicities and has also been found to be a risk factor for developing carcinogenesis and coronary heart diseases through experiments performed on animals. All this information needs to be communicated to the parents of the infected newborns, and special emphasis is on the point that ganciclovir is not efficient in reversing the established injury in the central nervous system [29]. The drug's toxicity is supposed to be monitored closely when long-term antiviral treatment is administered [30]. 

Speech or occupational treatment for children suffering from hearing loss should be provided. These services ensure that the children acquire critical social, linguistic, and communication skills. Children with this hearing impairment can also learn sign language and other alternative forms of communication, as well as how to use assistive listening technologies like cochlear implants and hearing aids. Children with hearing loss have a better chance of realising their full potential the earlier they begin receiving treatments. Cochlear implants and external hearing aids are beneficial in such cases. It is estimated that 2% of children with asymptomatic congenital CMV develop hearing loss severe enough to meet cochlear implantation candidacy criteria [31].

**Figure 1:**
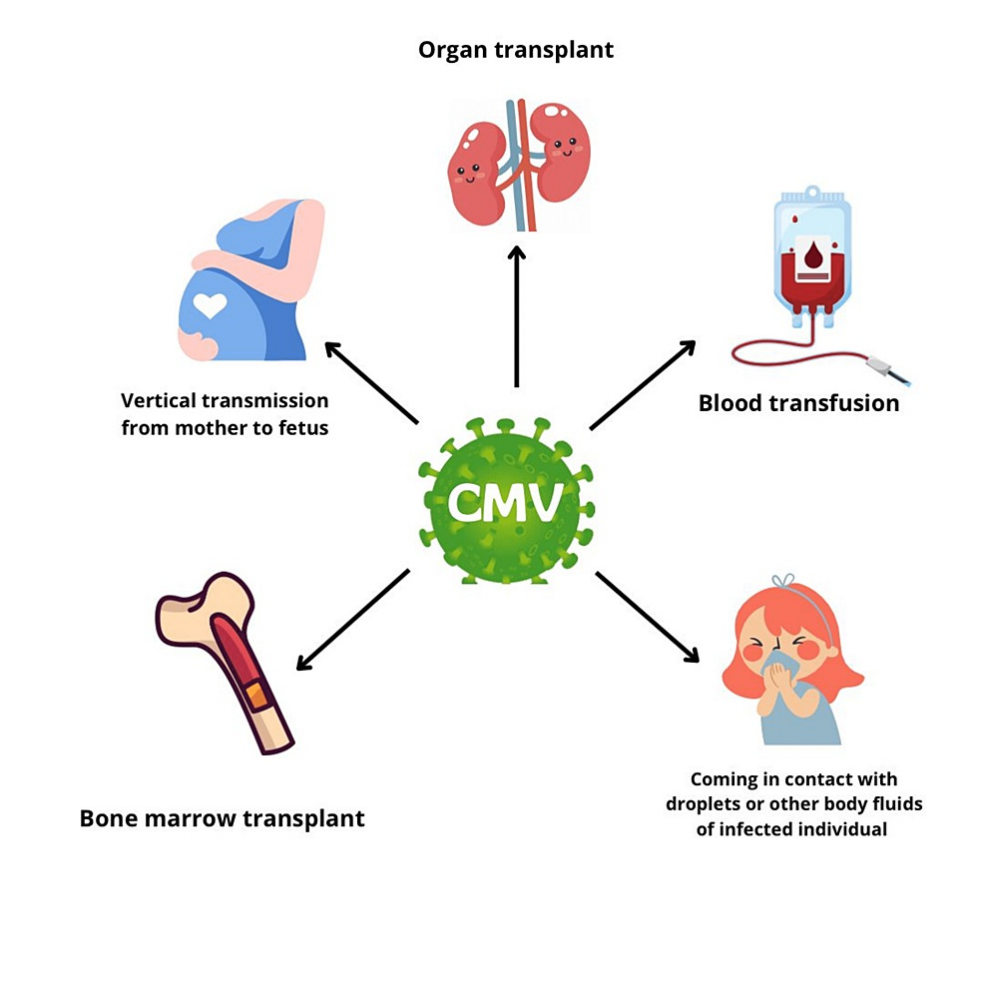
Common routes of transmission of cytomegalovirus (CMV) The image was created by the authors.

Vaccines for mothers, if found positive for CMV, are available to prevent the risk of the spread of infection. Maternal infection prevention primarily rests on behavioural interventions in the absence of effective vaccination efforts [32]. Compared to older children and adults, infants and young children are more prone to shedding the HCMV in body fluids like urine and saliva. Most pregnant women contract the CMV while caring for children at home or while working in daycare facilities. Educating the mothers at the time of pregnancy can stop the mother from contracting the CMV infection. However, because most women are unaware of the HCMV or how to prevent it, preventative opportunities are lost. Educating pregnant women can efficiently prevent the vertical transmission of the infection to the foetus and minimise congenital CMV. Although CMV infection can be spread through sexual contact, the main method of transmission for women of reproductive age is through contact with the urine and saliva of young infants. Effective behavioural strategies include not kissing small children, refraining from changing diapers and sharing food and drinks also, and washing hands as soon as possible after wiping their mouths and noses [33]. Effective behavioural prevention can stop the transmission of HCMV in seronegative women [34]. The causes of postpartum infections are multifaceted, such as exposure to maternal reproductive tract secretions, via breastmilk, and HCMV serological blood transfusion during delivery [35]. Women who are seropositive can also benefit from limiting their CMV exposure. HCMV infection, the commonest cause of congenital malformations, can result in complications with the CNS such as sensorineural hearing loss. The method by which HCMV enters the spiral ganglion neurons of the inner ear triggering the cascade of immunological responses resulting in spiral ganglion neurons cell apoptosis and destruction of the structure of the inner ear is the subject of considerable investigation at the moment. Ganciclovir and/or valganciclovir are still effective antiviral medications for HCMV infection. The common routes of CMV transmission have been depicted in Figure 1.

## Conclusions

Despite improvements in the treatment, clinically speaking, prenatal detection tools are still lacking. Serological testing is the main method used to determine whether a pregnant woman has a primary HCMV infection. The IgG affinity and the negative-to-positive seroconversion can be used to identify the mother's original infection. The placenta is a route by which HCMV can reach the fetus. PCR and amniocentesis for viral culture could be utilised to identify fetal HCMV infection. Because the majority of infected people have no symptoms, diagnosing newborn HCMV is challenging. The gold standard method for the diagnosis of infection caused due to the CMV is the isolation of the virus from saliva or urine. Currently, neonatal hearing screening is the main method used to detect symptomatic sensorineural hearing loss. Prenatal and neonatal HCMV screening is therefore crucial yet incomplete.

Unfortunately, there are no HCMV vaccinations available to protect against HCMV infection. HIV injection, however, has been proven in tests to be able to stop HCMV from infecting the foetus through the placenta. However, health counselling for women of reproductive age, which is also conducive to screening seronegative women, is the most efficient method of preventing HCMV vertical transmission. But once HCMV infection is discovered in pregnant women, there is neither an effective medication nor a strategy for limiting the risk of transmitting the infection to the foetus. Therefore, we support the use of behavioural preventative measures such as frequent hand washing, intervention, and screening during pregnancy, refraining from feeding and drinking with children, and avoiding kissing their mouths. Behaviour control has been found in a study to lower maternal CMV infection, therefore, shielding the babies from viral infection. Health education for women of childbearing age, newborn hearing screening, subsequent CMV PCR identification of symptomatic infants, and symptomatic infants with antiviral treatment are thought to be effective ways to lower the incidence of SNHL following HCMV infection. Further investigations are required to investigate the mechanism of SNHL caused due to the HCMV.
